# Meta-analysis of whole-exome sequencing data from two independent cohorts finds no evidence for rare variant enrichment in Parkinson disease associated loci

**DOI:** 10.1371/journal.pone.0239824

**Published:** 2020-10-01

**Authors:** Johannes Jernqvist Gaare, Gonzalo Nido, Christian Dölle, Paweł Sztromwasser, Guido Alves, Ole-Bjørn Tysnes, Kristoffer Haugarvoll, Charalampos Tzoulis

**Affiliations:** 1 Department of Neurology, Haukeland University Hospital, Bergen, Norway; 2 Department of Clinical Medicine, University of Bergen, Bergen, Norway; 3 Department of Clinical Science, University of Bergen, Bergen, Norway; 4 Computational Biology Unit, Institute of Informatics, University of Bergen, Bergen, Norway; 5 Department of Biostatistics and Translational Medicine, Medical University of Lodz, Lodz, Poland; 6 The Norwegian Centre for Movement Disorders and Department of Neurology, Stavanger University Hospital, Stavanger, Norway; 7 Department of Chemistry, Bioscience and Environmental Engineering, University of Stavanger, Stavanger, Norway; Universita degli Studi di Roma Tor Vergata, ITALY

## Abstract

Parkinson disease (PD) is a complex neurodegenerative disorder influenced by both environmental and genetic factors. While genome wide association studies have identified several susceptibility loci, many causal variants and genes underlying these associations remain undetermined. Identifying these is essential in order to gain mechanistic insight and identify biological pathways that may be targeted therapeutically. We hypothesized that gene-based enrichment of rare mutations is likely to be found within susceptibility loci for PD and may help identify causal genes. Whole-exome sequencing data from two independent cohorts were analyzed in tandem and by meta-analysis and a third cohort genotyped using the NeuroX-array was used for replication analysis. We employed collapsing methods (burden and the sequence kernel association test) to detect gene-based enrichment of rare, protein-altering variation within established PD susceptibility loci. Our analyses showed trends for three genes (*GALC*, *PARP9* and *SEC23IP*), but none of these survived multiple testing correction. Our findings provide no evidence of rare mutation enrichment in genes within PD-associated loci, in our datasets. While not excluding that rare mutations in these genes may influence the risk of idiopathic PD, our results suggest that, if such effects exist, much larger sequencing datasets will be required for their detection.

## Introduction

Parkinson disease (PD) is a complex disorder influenced by the crosstalk between genetic and environmental factors [1]. Monogenic causes account for a small fraction of cases, whereas the vast majority of patients have idiopathic disease. While genome-wide association studies (GWAS) have revealed several susceptibility *loci* for idiopathic PD, these collectively explain only a fraction of the disorder’s estimated heritability, and most have not been linked to pathways which can be targeted by therapies [2]. This is partly due to the uncertainty regarding which genes actually drive the GWAS signals.

The associated variants in GWAS are typically located in noncoding regions of the genome and assumed to be in linkage disequilibrium (LD) with causative variants in nearby genes [3]. Methods to identify candidate genes from GWAS range from simply choosing the closest gene to more sophisticated algorithms [4], but all are, in essence, inferential by nature. Next generation sequencing technologies have enabled us to investigate the impact of rare genetic variation, which is theorized to explain parts of the “missing heritability” in complex diseases [5]. In PD, rare variants have been implicated in sporadic disease both at the gene- [6] and pathway level [7,8]. Whether rare variants can explain GWAS signals in PD, remains, however, unknown.

We hypothesized that gene-based enrichment of rare, protein-altering variation is likely to be found in regions tagged by single nucleotide polymorphisms (SNPs) associated with PD in GWAS, and may help identify the causal genes driving these associations. To test our hypothesis, we selected genes with variants in LD with associated SNPs from the most recent GWAS meta-analysis[9], and tested for enrichment of rare, protein-altering variants in whole-exome sequencing data from two independent cohorts.

## Methods

### Cohorts and sequencing

The Norwegian whole-exome sequencing (WES) cohort comprised 191 patients with PD from the Norwegian ParkWest study [10] and 219 controls. The control group consisted of individuals with testis cancer (n = 167) and acoustic neuroma (n = 52) who had been recruited and examined at our hospital and had no clinical signs of neurodegenerative- or other neurological disorders. DNA was extracted from blood by routine procedures and sequenced at HudsonAlpha Institute for Biotechnology (Huntsville, Alabama) on the Illumina HiSeq platform using paired-end 100 bp sequencing and Roche-NimbleGen Sequence Capture EZ Exome v2 (173 controls) and v3 (all PD and 46 controls) capture kits. Reads were mapped to the hg19 (GRCh37) reference genome using BWA v0.6.2 [11], PCR duplicates removed with Picard v1.118 [12], and the alignment refined using Genome Analysis Toolkit (GATK) v3.3.0 [13] applying base quality score recalibration and realignment around indels recommended in the GATK Best Practices workflow [14,15]. Variants were called in all samples using GATK HaplotypeCaller [13] with default parameters. Next, Variant Quality Score Recalibration (VQSR) was performed using 99.9% sensitivity threshold [13]. The remaining variants were filtered against the intersection of capture targets (v2 and v3) using BEDtools [16] and VCFtools [17]. Variants with total depth below 10X were marked as unknown genotype (no-call) using BCFtools [18]. In addition, we used a cutoff of at least 6 reads supporting each variant (alternated allele). Indels were removed prior to downstream analyses. The depth distribution for all variants and variants of interest is shown in S1 Fig.

Additional whole-exome sequencing data was obtained from the Parkinson Progression Markers Initiative (PPMI) [19]. WES data was available from 640 individuals (459 cases and 181 controls). Control subjects were individuals without PD 30 years or older, without first degree relatives with PD. Sequencing was performed on the Illumina HiSeq 2500 platform using the Illumina Nextera Rapid Capture Expanded Exome Kit and paired-end 100 bp reads. Calling and alignment were performed by the PPMI. Indels were removed prior to variant quality control using VCFTools [17].

SNP-chip data was obtained from the International Parkinson’s Disease Genomics Consortium (IPDGC) (dbGaP Study Accession: phs000918.v1.p1). The dataset consisted of 11,402 individuals (5,540 cases and 5,862 controls) genotyped on the NeuroX array, comprising approximately 240,000 standard Illumina exome variants and 24,000 custom variants focusing on neurological diseases [20,21].

### Individual and variant quality control

Sequencing and genotype data were recoded into binary PLINK input format, and quality control of individual and SNP data was performed for all three cohorts separately. Individuals were excluded if they had an individual genotype missingness rate of > 2%, heterozygosity outside +/- 3 standard deviations (calculated for common and rare variants separately), cryptic relatedness (IBD > 0.2), conflicting sex assignment or non-European ancestry. Population stratification was studies using multi-dimensional scaling against the HapMap populations [22]. Variants were removed if they had a genotyping rate < 98%, different call rates in cases and controls (p > 0.02) or departure from the Hardy-Weinberg equilibrium (p < 10^−5^). Only autosomes were kept for downstream analyses. Principal component analysis was performed using Eigensoft [23,24] with standard filtering settings. ANOVA of the 10 first principal components was performed with the significance level set to p < 0.01. Significant principal components were included as covariates in all downstream analyses. Outside of the principal component analysis, all quality control procedures were performed using PLINK v1.90 [25] and R [26].

### Annotation and subset filtering

The datasets were annotated using ANNOVAR [27] according to the RefSeq gene transcripts, and variants classified as nonsynonymous, stop-gain, stop-loss or splicing were extracted for further analysis. Rare variants were defined as having a minor allele frequency (MAF) of < 1% in the non-Finnish European population in gnomAD [28].

### Selection of genes of interest

Genomic regions associated with PD where extracted from the largest and most recent, to date, meta-analysis of GWASes, which identified 90 SNPs associated with PD at genome-wide significance level [9]. We defined genes of interest as any gene containing a variant in LD within a 2 megabase window around any of these 90 SNPs, with the threshold of LD set to R^2^ > 0.5. If a variant in LD was localized in an intergenic region, the nearest gene was included. LD calculations were available from the supplementary material of the original study [9], and a total of 303 genes fit the inclusion criteria (S1 Table).

### Genetic association analyses

For each cohort, genes of interest were analyzed by two different tests: the burden test and the sequence kernel association test (SKAT) [29], using the SKAT R package v1.3.2.1 [30] with default settings. Statistically significant principal components, as determined by an ANOVA of the first 10 principal components with significance level cutoff set to p < 0.01, were added as covariates to all downstream analyses. Only genes with variants in both WES cohorts (ParkWest and PPMI) and at least two or more variants across cohorts were included. The meta-analysis was performed using the MetaSKAT R package v0.60 [31], using the same burden test and SKAT as described above in a meta-analysis framework. For the meta-analysis, we hypothesized that genetic effects should be homogenous across studies, meaning that the same mutation should have the same direction of effect in both cohorts. NeuroX was used as a replication cohort for the results from the WES analyses, and analyzed using the same methods. Only variants defined as rare were included. All p-values were corrected for multiple comparisons using FDR (Benjamini-Hochberg) [32].

### Gene set analyses

In addition to the single gene analyses, enrichment of genetic variation across all genes of interest was explored in a gene set analysis. Only rare variants were included, and the combined gene set was analyzed by burden and SKAT tests using the same methods and statistical tools as for the single gene analyses. A subset of loss-of-function (LoF) variants (containing only splicing, stop-loss and stop-gain variants) was extracted and similarly analyzed.

### Ethical considerations

These studies were approved by the Regional Committee for Medical and Health Research Ethics, Western Norway (REK 131/04), and all subjects gave written, informed consent. All research was performed in accordance with the relevant guidelines and regulations.

## Results

Using the inclusion criteria outlined previously, 168 genes of interest were analyzed in the single gene analyses, comprising a total of 543 rare nonsynonymous, stop-gain, stop-loss or splicing variants in the ParkWest cohort, and 1135 in the PPMI cohort. 160 of these genes were available for replication analysis in the NeuroX dataset, comprising a total of 1380 variants. For the gene set analysis, the number of included variants was 554 in the ParkWest, 1341 in the PPMI and 1534 in the NeuroX cohorts. A total of 14 LoF variants were identified in the ParkWest cohort, 17 in the PPMI cohort and 40 in the NeuroX cohort.

Gene-based analyses indicated three genes with nominally significant p-values (uncorrected p < 0.05) across multiple cohorts: *GALC*, *SEC23IP* and *PARP9*. However, no gene reached statistical significance surviving multiple testing correction in either of the cohorts or the meta-analysis (see S2 Table). Similarly, there were no statistically significant results in the gene set analyses (see S3 Table). The top results of the gene enrichment analyses, ranked by nominal p-value in the meta-analysis, are shown in the Tables 1 and 2.

**Table 1:** Top results for burden-based gene enrichment analyses.

Gene	ParkWest			PPMI			Meta			NeuroX		
	Variants	P-value	FDR	Variants	P-value	FDR	Variants	P-value	FDR	Variants	P-value	FDR
SEC23IP	3	0.0276	0.9035	9	0.0332	0.9191	11	0.0040	0.6669	10	0.6819	0.9037
PARP9	3	0.0819	0.9035	9	0.0730	0.9191	11	0.0110	0.7058	7	0.0908	0.6353
GALC	4	0.8111	0.9035	5	0.0032	0.5335	8	0.0210	0.7058	7	0.2607	0.8180
NFKB2	1	0.0180	0.9035	7	0.2543	0.9191	7	0.0333	0.7058	6	0.7477	0.9037
ATP2A1	2	0.0518	0.9035	10	0.2232	0.9191	12	0.0416	0.7058	7	0.6897	0.9037
PBXIP1	1	0.3142	0.9035	8	0.0656	0.9191	9	0.0457	0.7058	10	0.6148	0.9037
CASR	1	0.6461	0.9035	4	0.0276	0.9191	4	0.0457	0.7058	6	0.5906	0.9037
ITGA8	2	0.1473	0.9035	10	0.1905	0.9191	12	0.0533	0.7058	18	0.6969	0.9037
VPS13C	26	0.2533	0.9035	24	0.1276	0.9191	42	0.0557	0.7058	49	0.0636	0.5420
CTSB	3	0.9743	0.9801	15	0.0279	0.9191	17	0.0688	0.7058	10	0.4903	0.9037

Genes are ranked by p-value in the meta-analysis. The FDR-column contains p-values after applying false discovery rate-correction.

**Table 2:** Top results from SKAT-based gene enrichment analyses.

Gene	ParkWest			PPMI			Meta			NeuroX		
	Variants	P-value	FDR	Variants	P-value	FDR	Variants	P-value	FDR	Variants	P-value	FDR
CASR	1	0.6461	0.9008	4	0.0012	0.2089	4	0.0029	0.4900	6	0.7926	0.9880
PARP9	3	0.1087	0.9008	9	0.1820	0.9393	11	0.0215	0.8873	7	0.0311	0.5528
GALC	4	0.0460	0.9008	5	0.0366	0.9393	8	0.0311	0.8873	7	0.8539	0.9880
NFKB2	1	0.0180	0.9008	7	0.9714	0.9944	7	0.0381	0.8873	6	0.2684	0.9257
SEC23IP	3	0.1313	0.9008	9	0.1113	0.9393	11	0.0469	0.8873	10	0.3037	0.9320
SCARB2	2	0.1983	0.9008	3	0.4048	0.9393	4	0.0829	0.8873	11	0.5043	0.9613
BTNL2	1	0.7423	0.9008	8	0.1152	0.9393	9	0.1085	0.8873	14	0.3768	0.9494
CTSB	3	0.4239	0.9008	15	0.1788	0.9393	17	0.1153	0.8873	10	0.0664	0.7225
PAM	5	0.1454	0.9008	13	0.2638	0.9393	17	0.1188	0.8873	11	0.8316	0.9880
TUFM	1	0.3609	0.9008	2	0.5208	0.9393	2	0.1205	0.8873	1	0.1826	0.9257

Genes are ranked by p-value in the meta-analysis. The FDR-column contains p-values after applying false discovery rate-correction.

## Discussion

Our analyses revealed no statistically significant enrichment of rare variants in genes implicated by previous GWAS in PD. Three genes (*GALC*, *SEC23IP* and *PARP9*) showed trends across multiple cohorts, but none survived multiple testing correction. Nalls et al [9] conducted rare variant burden analysis for *SEC23IP* finding no enrichment signal. Thus, it is highly unlikely that *SEC23IP* is involved in PD. The variant tagging *PARP9* (rs55961674) is a weak expression quantitative trait loci (eQTL) for *PARP9* in some tissues (nerve and thyroid) [33]. However, it is also a strong splicing QTL (sQTL) for *KPNA1*, suggesting that this is a more likely candidate gene. Finally, the variant tagging *GALC* (rs979812) is a strong eQTL for *GALC*, supporting a potential role in PD [33]. *GALC* encodes the enzyme galactocerebrosidase, and mutations in this gene cause Krabbe disease, a lysosomal storage disorder [34]. Current evidence suggests that lysosomal dysfunction plays a key role in PD [35], and rare mutations in a broad range of genes causing lysosomal storage disorders have been associated with PD [7]. Mutations of *GBA* in particular, the gene encoding the enzyme glucosylcerebrosidase that carries out a very similar reaction to that of galactocerebrosidase, are the most common genetic risk factor for PD and this association is driven by both common [36] and rare variants [37]. A role for *GALC* in α-synucleinopathies is therefore not farfetched [38].

Taken together, our findings provide no evidence of rare mutation enrichment in PD GWAS loci, in our datasets. These results do not support our initial hypothesis that gene-based enrichment of rare mutations can be helpful in identifying causal genes in PD-associated loci. It should be stressed that these findings do not disprove the hypothesis that rare mutations in these genes may influence the risk of idiopathic PD. They do, however, suggest that if such effects exist, much larger sequencing datasets will be required for their detection.

A few studies with similar approaches to ours have previously been published, using older GWAS data. Foo et al [39] probed 39 genes implicated in PD by GWAS and described enrichment of rare missense variation in *LRRK2*. Sandor et al [40] investigated 329 genes located within GWAS loci, and detected a possible enrichment of missense variation, including both common and rare mutations in their analysis, across the complete gene set. Finally, Jansen et al [41] used a Prix fixe strategy to select one candidate gene per GWAS locus, and detected rare variation association signals in *LRRK2*, *STBD1* and *SPATA19*. While we could not replicate enrichment for any of these genes in our datasets, it should be noted that our sample size (n = 1050) is smaller than that of Jansen et al [6,41].

In addition to rare variant enrichment analyses, several other methodologies have been employed to nominate causal genes from GWAS loci. eQTL studies integrate genotype and gene expression date, to identify genes whose expression is regulated by PD associated SNPs [42–46]. The effect of non-coding genetic variation on splicing of pre-mRNA (splicing QTLs or sQTLs) has also recently been highlighted and used to further explore possible causal genes in PD [47]. Finally, epigenetic quantitative trait loci, such as DNA methylation (mQTL), have also been used in combination with GWAS and eQTL data with variable success [48].

PD is a complex disease of heterogeneous etiology. While there is a clear genetic component, as evidenced by twin studies [49], known risk loci are primarily common mutations which, collectively, only explain a fraction of the total estimated heritability [9]. As for other complex disorders, much of the unexplained heritability is believed to be caused by rare variants [50]. Multiple studies have linked common mutations, either through the use of polygenic risk scores [51] or machine learning algorithms [42], to motor progression and cognitive decline. In addition, common genetic variation has also been shown to impact drug responsiveness in PD [52]. Similar applications of rare variants could potentially increase the predictive precision of these models and provide clinicians with a powerful tool to individualize treatment and follow-up for PD patients.

In conclusion, our results indicate that rare variant enrichment alone is unlikely to be helpful in identifying causal risk genes for PD in small to moderately sized cohorts. Larger studies are needed to determine if rare variant enrichment with small effect sizes are present in these genes. Future studies will likely need to integrate multiple types of data, including GWAS, sequencing and various forms of QTL analyses as well as functional experiments in order to better characterize the effects of rare coding variation in PD and identify novel genes and biological pathways.

## Supporting information

S1 Fig Variant depth distribution. A) Depth distribution of all variants called across all samples. B) Depth distribution for the subset of variants called within the predefined regions of interest across all samples. Red bars represent heterozygous variants (0/1), and blue bars represent homozygous (1/1) variants. The vertical dashed line represents the cutoff of minimum 10 reads employed in the analyses.
(PDF)

S1 Table Genes of interest. (PDF)

S2 Table Complete results from gene-based rare variant enrichment analyses. (PDF)

S3 Table Gene set analyses. (PDF)

## References

[1] Deng H, Wang P, Jankovic J. The genetics of Parkinson disease. Ageing Res Rev. 2018;42:72–85. Epub 2017/12/31. 10.1016/j.arr.2017.12.007 . 29288112

[2] Keller MF, Saad M, Bras J, Bettella F, Nicolaou N, Simon-Sanchez J, et al Using genome-wide complex trait analysis to quantify ‘missing heritability’ in Parkinson’s disease. Hum Mol Genet. 2012;21(22):4996–5009. 10.1093/hmg/dds335 . 22892372 PMC3576713

[3] Zhang X, Bailey SD, Lupien M. Laying a solid foundation for Manhattan—'setting the functional basis for the post-GWAS era'. Trends Genet. 2014;30(4):140–9. Epub 2014/03/26. 10.1016/j.tig.2014.02.006 . 24661571 PMC4026049

[4] Tasan M, Musso G, Hao T, Vidal M, MacRae CA, Roth FP. Selecting causal genes from genome-wide association studies via functionally coherent subnetworks. Nat Methods. 2015;12(2):154–9. Epub 2014/12/23. 10.1038/nmeth.3215 . 25532137 PMC4480866

[5] Bomba L, Walter K, Soranzo N. The impact of rare and low-frequency genetic variants in common disease. Genome Biol. 2017;18(1):77 Epub 2017/04/30. 10.1186/s13059-017-1212-4 . 28449691 PMC5408830

[6] Jansen IE, Ye H, Heetveld S, Lechler MC, Michels H, Seinstra RI, et al Discovery and functional prioritization of Parkinson’s disease candidate genes from large-scale whole exome sequencing. Genome Biol. 2017;18(1):22 10.1186/s13059-017-1147-9 . 28137300 PMC5282828

[7] Robak LA, Jansen IE, van Rooij J, Uitterlinden AG, Kraaij R, Jankovic J, et al Excessive burden of lysosomal storage disorder gene variants in Parkinson’s disease. Brain. 2017;140(12):3191–203. Epub 2017/11/16. 10.1093/brain/awx285 . 29140481 PMC5841393

[8] Gaare JJ, Nido GS, Sztromwasser P, Knappskog PM, Dahl O, Lund-Johansen M, et al Rare genetic variation in mitochondrial pathways influences the risk for Parkinson’s disease. Mov Disord. 2018;33(10):1591–600. Epub 2018/09/27. 10.1002/mds.64 . 30256453 PMC6282592

[9] Nalls MA, Blauwendraat C, Vallerga CL, Heilbron K, Bandres-Ciga S, Chang D, et al Expanding Parkinson’s disease genetics: novel risk loci, genomic context, causal insights and heritable risk. bioRxiv. 2019:388165 10.1101/388165

[10] Alves G, Muller B, Herlofson K, HogenEsch I, Telstad W, Aarsland D, et al Incidence of Parkinson’s disease in Norway: the Norwegian ParkWest study. J Neurol Neurosurg Psychiatry. 2009;80(8):851–7. 10.1136/jnnp.2008.168211 . 19246476

[11] Li H, Durbin R. Fast and accurate short read alignment with Burrows-Wheeler transform. Bioinformatics. 2009;25(14):1754–60. 10.1093/bioinformatics/btp324 . 19451168 PMC2705234

[12] http://broadinstitute.github.io/picard [cited 2016 December].

[13] McKenna A, Hanna M, Banks E, Sivachenko A, Cibulskis K, Kernytsky A, et al The Genome Analysis Toolkit: a MapReduce framework for analyzing next-generation DNA sequencing data. Genome Res. 2010;20(9):1297–303. 10.1101/gr.107524.110 . 20644199 PMC2928508

[14] DePristo MA, Banks E, Poplin R, Garimella KV, Maguire JR, Hartl C, et al A framework for variation discovery and genotyping using next-generation DNA sequencing data. Nat Genet. 2011;43(5):491–8. 10.1038/ng.806 . 21478889 PMC3083463

[15] Van der Auwera GA, Carneiro MO, Hartl C, Poplin R, Del Angel G, Levy-Moonshine A, et al From FastQ data to high confidence variant calls: the Genome Analysis Toolkit best practices pipeline. Curr Protoc Bioinformatics. 2013;43:11 0 1–33. 10.1002/0471250953.bi1110s43 . 25431634 PMC4243306

[16] Quinlan AR. BEDTools: The Swiss-Army Tool for Genome Feature Analysis. Curr Protoc Bioinformatics. 2014;47:11 2 1–34. 10.1002/0471250953.bi1112s47 . 25199790 PMC4213956

[17] Danecek P, Auton A, Abecasis G, Albers CA, Banks E, DePristo MA, et al The variant call format and VCFtools. Bioinformatics. 2011;27(15):2156–8. 10.1093/bioinformatics/btr330 . 21653522 PMC3137218

[18] Li H. A statistical framework for SNP calling, mutation discovery, association mapping and population genetical parameter estimation from sequencing data. Bioinformatics. 2011;27(21):2987–93. 10.1093/bioinformatics/btr509 . 21903627 PMC3198575

[19] Parkinson Progression Marker I. The Parkinson Progression Marker Initiative (PPMI). Prog Neurobiol. 2011;95(4):629–35. 10.1016/j.pneurobio.2011.09.005 . 21930184 PMC9014725

[20] Nalls MA, Pankratz N, Lill CM, Do CB, Hernandez DG, Saad M, et al Large-scale meta-analysis of genome-wide association data identifies six new risk loci for Parkinson’s disease. Nat Genet. 2014;46(9):989–93. Epub 2014/07/30. 10.1038/ng.3043 . 25064009 PMC4146673

[21] Nalls MA, Bras J, Hernandez DG, Keller MF, Majounie E, Renton AE, et al NeuroX, a fast and efficient genotyping platform for investigation of neurodegenerative diseases. Neurobiol Aging. 2015;36(3):1605 e7–12. Epub 2014/12/03. 10.1016/j.neurobiolaging.2014.07.028 . 25444595 PMC4317375

[22] International HapMap C. The International HapMap Project. Nature. 2003;426(6968):789–96. Epub 2003/12/20. 10.1038/nature02168 . 14685227

[23] Price AL, Patterson NJ, Plenge RM, Weinblatt ME, Shadick NA, Reich D. Principal components analysis corrects for stratification in genome-wide association studies. Nat Genet. 2006;38(8):904–9. 10.1038/ng1847 . 16862161

[24] Patterson N, Price AL, Reich D. Population structure and eigenanalysis. PLoS Genet. 2006;2(12):e190 Epub 2006/12/30. 10.1371/journal.pgen.0020190 . 17194218 PMC1713260

[25] Chang CC, Chow CC, Tellier LC, Vattikuti S, Purcell SM, Lee JJ. Second-generation PLINK: rising to the challenge of larger and richer datasets. Gigascience. 2015;4:7 10.1186/s13742-015-0047-8 . 25722852 PMC4342193

[26] The R Project for Statistical Computing. www.r-project.org.

[27] Wang K, Li M, Hakonarson H. ANNOVAR: functional annotation of genetic variants from high-throughput sequencing data. Nucleic Acids Res. 2010;38(16):e164 10.1093/nar/gkq603 . 20601685 PMC2938201

[28] Lek M, Karczewski KJ, Minikel EV, Samocha KE, Banks E, Fennell T, et al Analysis of protein-coding genetic variation in 60,706 humans. Nature. 2016;536(7616):285–91. Epub 2016/08/19. 10.1038/nature19057 . 27535533 PMC5018207

[29] Wu MC, Lee S, Cai T, Li Y, Boehnke M, Lin X. Rare-variant association testing for sequencing data with the sequence kernel association test. Am J Hum Genet. 2011;89(1):82–93. 10.1016/j.ajhg.2011.05.029 . 21737059 PMC3135811

[30] Lee S, Fuchsberger C, Kim S, Scott L. An efficient resampling method for calibrating single and gene-based rare variant association analysis in case-control studies. Biostatistics. 2016;17(1):1–15. 10.1093/biostatistics/kxv033 . 26363037 PMC4692986

[31] Lee S, Teslovich TM, Boehnke M, Lin X. General framework for meta-analysis of rare variants in sequencing association studies. Am J Hum Genet. 2013;93(1):42–53. Epub 2013/06/19. 10.1016/j.ajhg.2013.05.010 . 23768515 PMC3710762

[32] Benjamini Y, Hochberg Y. Controlling the False Discovery Rate: A Practical and Powerful Approach to Multiple Testing. Journal of the Royal Statistical Society Series B (Methodological). 1995;57(1):289–300. 10.2307/2346101

[33] Consortium GTEx. The Genotype-Tissue Expression (GTEx) project. Nature genetics. 2013;45(6):580–5. 10.1038/ng.2653 . 23715323 PMC4010069

[34] Ferreira CR, Gahl WA. Lysosomal storage diseases. Transl Sci Rare Dis. 2017;2(1–2):1–71. Epub 2017/11/21. 10.3233/TRD-160005 . 29152458 PMC5685203

[35] Moors T, Paciotti S, Chiasserini D, Calabresi P, Parnetti L, Beccari T, et al Lysosomal Dysfunction and alpha-Synuclein Aggregation in Parkinson’s Disease: Diagnostic Links. Mov Disord. 2016;31(6):791–801. Epub 2016/03/01. 10.1002/mds.26562 . 26923732

[36] Gegg ME, Schapira AHV. The role of glucocerebrosidase in Parkinson disease pathogenesis. FEBS J. 2018 Epub 2018/02/01. 10.1111/febs.14393 . 29385658

[37] Davis AA, Andruska KM, Benitez BA, Racette BA, Perlmutter JS, Cruchaga C. Variants in GBA, SNCA, and MAPT influence Parkinson disease risk, age at onset, and progression. Neurobiol Aging. 2016;37:209 e1–e7. Epub 2015/11/26. 10.1016/j.neurobiolaging.2015.09.014 . 26601739 PMC4688052

[38] Marshall MS, Bongarzone ER. Beyond Krabbe’s disease: The potential contribution of galactosylceramidase deficiency to neuronal vulnerability in late-onset synucleinopathies. J Neurosci Res. 2016;94(11):1328–32. Epub 2016/09/18. 10.1002/jnr.23751 . 27638614 PMC5027968

[39] Foo JN, Tan LC, Liany H, Koh TH, Irwan ID, Ng YY, et al Analysis of non-synonymous-coding variants of Parkinson’s disease-related pathogenic and susceptibility genes in East Asian populations. Human Molecular Genetics. 2014;23(14):3891–7. 10.1093/hmg/ddu086 24565865

[40] Sandor C, Honti F, Haerty W, Szewczyk-Krolikowski K, Tomlinson P, Evetts S, et al Whole-exome sequencing of 228 patients with sporadic Parkinson’s disease. Sci Rep. 2017;7:41188 10.1038/srep41188 . 28117402 PMC5259721

[41] Jansen IE, Gibbs JR, Nalls MA, Price TR, Lubbe S, van Rooij J, et al Establishing the role of rare coding variants in known Parkinson’s disease risk loci. Neurobiol Aging. 2017;59:220 e11–e18. Epub 2017/09/05. 10.1016/j.neurobiolaging.2017.07.009 . 28867149 PMC8722270

[42] Latourelle JC, Beste MT, Hadzi TC, Miller RE, Oppenheim JN, Valko MP, et al Large-scale identification of clinical and genetic predictors of motor progression in patients with newly diagnosed Parkinson’s disease: a longitudinal cohort study and validation. The Lancet Neurology. 2017;16(11):908–16. 10.1016/S1474-4422(17)30328-9 28958801 PMC5693218

[43] Trabzuni D, Ryten M, Emmett W, Ramasamy A, Lackner KJ, Zeller T, et al Fine-Mapping, Gene Expression and Splicing Analysis of the Disease Associated LRRK2 Locus. PLOS ONE. 2013;8(8):e70724 10.1371/journal.pone.0070724 23967090 PMC3742662

[44] Trabzuni D, Wray S, Vandrovcova J, Ramasamy A, Walker R, Smith C, et al MAPT expression and splicing is differentially regulated by brain region: relation to genotype and implication for tauopathies. Human Molecular Genetics. 2012;21(18):4094–103. 10.1093/hmg/dds238 22723018 PMC3428157

[45] Pierce S, Coetzee GA. Parkinson’s disease-associated genetic variation is linked to quantitative expression of inflammatory genes. PLOS ONE. 2017;12(4):e0175882 10.1371/journal.pone.0175882 28407015 PMC5391096

[46] Murthy MN, Blauwendraat C, Guelfi S, Hardy J, Lewis PA, Trabzuni D, et al Increased brain expression of GPNMB is associated with genome wide significant risk for Parkinson’s disease on chromosome 7p15.3. neurogenetics. 2017;18(3):121–33. 10.1007/s10048-017-0514-8 28391543 PMC5522530

[47] Li YI, Wong G, Humphrey J, Raj T. Prioritizing Parkinson’s disease genes using population-scale transcriptomic data. Nature Communications. 2019;10(1):994 10.1038/s41467-019-08912-9 30824768 PMC6397174

[48] Kia DA, Zhang D, Guelfi S, Manzoni C, Hubbard L, Reynolds RH, et al Integration of eQTL and Parkinson’s disease GWAS data implicates 11 disease genes. bioRxiv. 2019:627216 10.1101/627216

[49] Wirdefeldt K, Gatz M, Reynolds CA, Prescott CA, Pedersen NL. Heritability of Parkinson disease in Swedish twins: a longitudinal study. Neurobiol Aging. 2011;32(10):1923 e1–8. Epub 2011/04/13. 10.1016/j.neurobiolaging.2011.02.017 . 21482443 PMC4452894

[50] Manolio TA, Collins FS, Cox NJ, Goldstein DB, Hindorff LA, Hunter DJ, et al Finding the missing heritability of complex diseases. Nature. 2009;461(7265):747–53. Epub 2009/10/09. 10.1038/nature08494 . 19812666 PMC2831613

[51] Paul KC, Schulz J, Bronstein JM, Lill CM, Ritz BR. Association of Polygenic Risk Score With Cognitive Decline and Motor Progression in Parkinson Disease. JAMA Neurology. 2018;75(3):360–6. 10.1001/jamaneurol.2017.4206 29340614 PMC5885856

[52] Miller NS, Chou KL, Bohnen NI, Müller MLTM, Seidler RD. Dopaminergic polymorphisms associated with medication responsiveness of gait in Parkinson’s disease. Parkinsonism & related disorders. 2018;48:54–60. Epub 2017/12/12. 10.1016/j.parkreldis.2017.12.010 . 29249680 PMC5826846

