# Supplementary material for: Meta-analysis of whole-exome sequencing data from two independent cohorts finds no evidence for rare variant enrichment in Parkinson disease associated loci
Source: PLoS One. 2020 Oct 1;15(10):e0239824. doi: 10.1371/journal.pone.0239824 (PMC7529297; doi:10.1371/journal.pone.0239824)
Supplement: S1 Fig — A) Depth distribution of all variants called across all samples. B) Depth distribution for the subset of variants called within the predefined regions of interest across all samples. Red bars represent heterozygous variants (0/1), and blue bars represent homozygous (1/1) variants. The vertical dashed line represents the cutoff of minimum 10 reads employed in the analyses. (PDF) [file pone.0239824.s001.pdf]

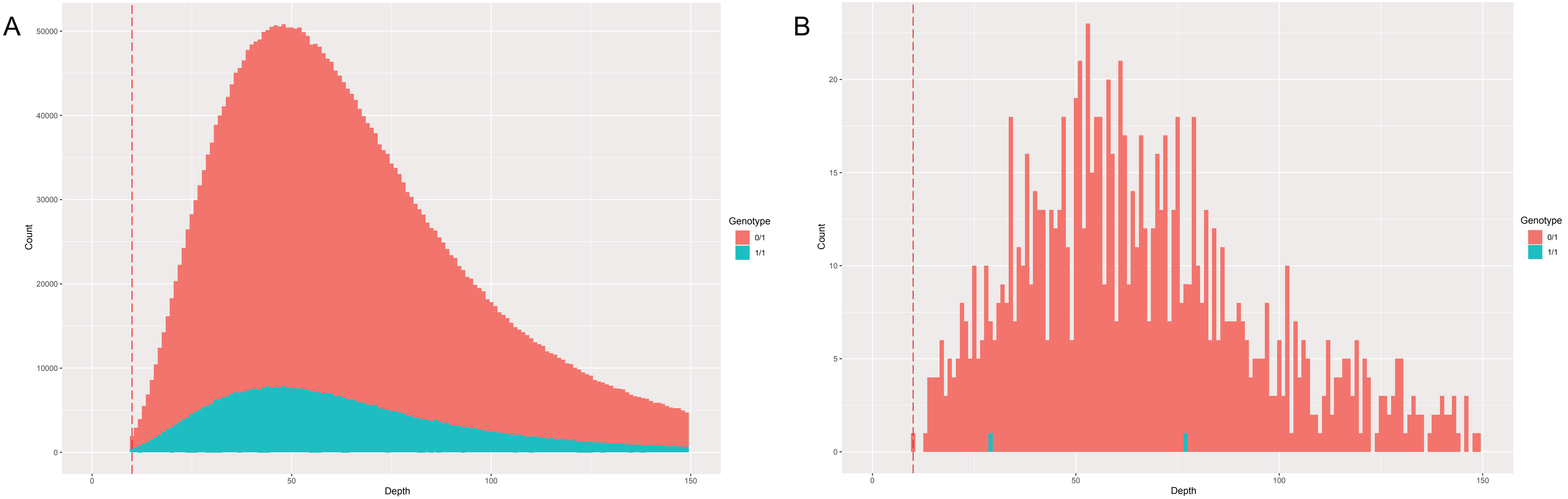

S1 Figure. Variant depth distribution. A) Depth distribution of all variants called across all samples. B) Depth distribution for the subset of variants called within the predefined regions of interest across all samples. Red bars represent heterozygous variants (0/1), and blue bars represent homozygous (1/1) variants. The vertical dashed line represents the cutoff of minimum 10 reads employed in the analyses.
