# Supplementary material for: Meta-analysis of whole-exome sequencing data from two independent cohorts finds no evidence for rare variant enrichment in Parkinson disease associated loci
Source: PLoS One. 2020 Oct 1;15(10):e0239824. doi: 10.1371/journal.pone.0239824 (PMC7529297; doi:10.1371/journal.pone.0239824)
Supplement: S1 Table — (PDF) [file pone.0239824.s002.pdf]

S1 Table - Genes of interest

AMPD3	DALRD3	HIST1H2AK	MAL	PROM2	TIAL1
ANKRD20A8P	DCAF12	HIST1H2AM	MALSU1	PROX2	TMEM163
AREL1	DCAF16	HIST1H2BL	MAP4K4	PRSS3	TMEM175
ARID2	DCUN1D1	HIST1H2BM	MBNL2	PRSS53	TMEM248
ARIH2	DDX46	HIST1H2BN	MCCC1	PSD	TNFSF12
ARIH2OS	DEPDC1B	HIST1H2BO	MED12L	PTENP1	TOB2P1
ART3	DGKQ	HIST1H3H	MED13	QARS	TOX3
ASH1L	DLG2	HIST1H3J	MEX3C	QRICH1	TPST1
ASL	DLST	HIST1H4J	MIPOL1	RAB29	TRIM10
ASXL3	DNAH17	HIST1H4L	MIR146B	RABEP2	TRIM15
ATP2A1	DPM3	HLA-DQA1	MIR4697HG	RABGEF1	TRIM26
ATP6V0A1	DTX3L	HLA-DQA2	MLX	RETREG3	TRIM31
ATXN2L	DYRK1A	HLA-DQB1	MMRN1	RIMS1	TRIM40
B3GALNT1	EEF1AKNMT	HLA-DRA	MRPS5	RIT2	TSBP1
BAG3	EGR3	HLA-DRB5	MTRNR2L8	RNF141	TUBG1
BCL7C	EIF3C	HLA-DRB6	NAGLU	RNF39	TUBG2
BIN3	ELOVL3	HSD17B1	NCAPG	RPS12	TUFM
BRIP1	ELOVL7	HSD3B7	NCKIPSD	RPS6KL1	TXNDC15
BST1	ERCC8	IGF2BP3	NDUFAF2	SAR1B	TYW1
BTNL2	FAM162A	IGSF9B	NDUFAF3	SATB1	UBAP2
C3orf84	FAM171A2	INPP5F	NEK1	SBDS	UBE2R2
C5orf24	FAM184B	INTS2	NFATC2IP	SCAF11	UBQLN4
C5orf30	FAM47E	INTS4P2	NFKB2	SCARB2	UBTF
C8orf58	FAM47E-STBD1	IP6K2	NMD3	SEC23IP	USP19
CAB39L	FAM49B	ITGA2B	NOD2	SEC24A	USP4
CAMK2D	FBRSL1	ITGA8	NOL4	SETD1A	VAMP4
CAMLG	FBXL19	ITPKB	NOLC1	SGF29	VKORC1
CASC16	FBXL19-AS1	JADE2	NUCKS1	SH2B1	VKORC1L1
CASR	FCF1	KCNIP3	NUPL2	SH3GL2	VPS13C
CATSPER3	FCGR2A	KCNN3	NUPR1	SH3RF1	WDHD1
CAVIN1	FDFT1	KCNS3	OR2B2	SIPA1L2	WDR5B
CCAR2	FGF20	KLHDC8B	OR2B6	SLC18B1	WDR6
CCDC36	FOXA1	KLHL7	P2RY12	SLC25A20	WNT3
CCDC58	FYN	KLHL7-DT	P4HTM	SLC25A21-AS1	YLPM1
CCDC62	GAK	KPNA1	PAM	SLC26A1	ZBTB4
CCDC71	GALC	KRTCAP2	PARP9	SLC45A3	ZBTB7B
CCT6P1	GBAP1	LAMB2	PART1	SLC4A1	ZKSCAN8
CD19	GBF1	LAMB2P1	PBXIP1	SLC50A1	ZNF165
CHD9	GCH1	LAT	PCBD2	SMIM15	ZNF192P1
CHRNB1	GIN1	LCORL	PDLIM2	SNCA	ZNF2
CLCN3	GPATCH8	LINC00174	PGF	SNCA-AS1	ZNF514
COASY	GPNMB	LINC00693	PGS1	SNX20	ZNF629
CPLX1	GPR65	LINC01012	PITX3	SPNS1	ZNF646
CRCP	GRN	LINC01146	PLEKHH3	SPPL2B	ZNF668
CRHR1	GS1-124K5.11	LINC02067	PMS2P4	SPTSSB	ZNRD1ASP

CRLS1	GTF2IRD1P1	LOC100131289	PMVK	STK39	ZSCAN12P1
CSTA	GUSB	LOC339862	POLR2A	STX1B	ZSCAN16
CTF1	HCG17	LOC441242	PPIP5K2	STX4	ZSCAN16-AS1
CTSB	HCG23	LOC442028	PPM1L	SULT1A1	
CUEDC2	HIP1R	LRRK2	PPP1R11	SYT17	
CYLD	HIST1H1B	LRRN4	PRKAR2A	SYT4	
