# Supplementary material for: Meta-analysis of whole-exome sequencing data from two independent cohorts finds no evidence for rare variant enrichment in Parkinson disease associated loci
Source: PLoS One. 2020 Oct 1;15(10):e0239824. doi: 10.1371/journal.pone.0239824 (PMC7529297; doi:10.1371/journal.pone.0239824)
Supplement: S2 Table — (PDF) [file pone.0239824.s003.pdf]

Supplementary Table S4 - Complete results from gene-based rare variant enrichment analyses

	ParkWest					PPMI					Meta					NeuroX				
**Gene**	Variants	Burden P	Burden P FDR	SKAT P	SKAT P FDR	Variants	Burden P	Burden P FDR	SKAT P	SKAT P FDR	Variants	Burden P	Burden P FDR	SKAT P	SKAT P FDR	Variants	Burden P	Burden P FDR	SKAT P	SKAT P FDR
AMPD3	6	0.5095	0.9035	0.2603	0.9008	13	0.8850	0.9470	0.3910	0.9393	16	0.6672	0.9054	0.3171	0.8873	14	0.5096	0.9037	0.3784	0.9494
AREL1	4	0.4650	0.9035	0.1691	0.9008	9	0.4811	0.9191	0.4540	0.9393	11	0.2819	0.7624	0.9029	0.9363	10	0.1500	0.6920	0.2398	0.9257
ARID2	4	0.1810	0.9035	0.3703	0.9008	8	0.8994	0.9503	0.7050	0.9611	10	0.3311	0.8061	0.8229	0.9060	11	0.7805	0.9037	0.5172	0.9736
ART3	3	0.1659	0.9035	0.9133	0.9487	2	0.3375	0.9191	0.7387	0.9611	5	0.0772	0.7058	0.6508	0.9051	9	0.7312	0.9037	0.0028	0.2205
ASH1L	4	0.8018	0.9035	0.0130	0.9008	18	0.6706	0.9191	0.8283	0.9611	21	0.6128	0.9054	0.3764	0.8873	20	0.7962	0.9037	0.8163	0.9880
ASL	2	0.3065	0.9035	0.4198	0.9008	6	0.5804	0.9191	0.5672	0.9393	7	0.9348	0.9800	0.6127	0.8873	9	0.7206	0.9037	0.8787	0.9880
ASXL3	7	0.8004	0.9035	0.7121	0.9008	20	0.8425	0.9191	0.7757	0.9611	24	0.7670	0.9406	0.6097	0.8873	22	0.7389	0.9037	0.2893	0.9257
ATP2A1	2	0.0518	0.9035	0.0518	0.9008	10	0.2232	0.9191	0.8880	0.9643	12	0.0416	0.7058	0.5010	0.8873	7	0.6897	0.9037	0.5593	0.9880
ATP6V0A1	3	0.9673	0.9790	0.2239	0.9008	9	0.5842	0.9191	0.3461	0.9393	10	0.8429	0.9605	0.7310	0.9051	9	0.6683	0.9037	0.7125	0.9880
ATXN2L	3	0.8098	0.9035	0.7619	0.9008	4	0.2026	0.9191	0.8638	0.9611	7	0.6171	0.9054	0.7247	0.9051	4	0.8912	0.9381	0.9598	0.9975
B3GALNT1	2	0.7441	0.9035	0.8298	0.9056	3	0.3756	0.9191	0.8113	0.9611	5	0.3763	0.8659	0.7472	0.9051	NA	NA	NA	NA	NA
BAG3	3	0.5142	0.9035	0.9375	0.9488	7	0.8397	0.9191	0.4606	0.9393	10	0.5957	0.9054	0.7474	0.9051	5	0.8193	0.9037	0.6422	0.9880
BIN3	3	0.7964	0.9035	0.5692	0.9008	1	0.6384	0.9191	0.6384	0.9393	4	0.9450	0.9800	0.6106	0.8873	3	0.0057	0.2263	0.0139	0.5158
BRIP1	8	0.1712	0.9035	0.2273	0.9008	10	0.1897	0.9191	0.4671	0.9393	14	0.9994	0.9994	0.2597	0.8873	13	0.4224	0.9037	0.2803	0.9257
BST1	3	0.7817	0.9035	0.8836	0.9377	4	0.2026	0.9191	0.8638	0.9611	7	0.2593	0.7498	0.8305	0.9060	5	0.5553	0.9037	0.2403	0.9257
BTNL2	1	0.7423	0.9035	0.7423	0.9008	8	0.1425	0.9191	0.1152	0.9393	9	0.1122	0.7058	0.1085	0.8873	14	0.7600	0.9037	0.3768	0.9494
C3orf84	1	0.7423	0.9035	0.7423	0.9008	1	0.1384	0.9191	0.1384	0.9393	2	0.7217	0.9054	0.1845	0.8873	1	0.2521	0.8180	0.2521	0.9257
C5orf30	1	0.7196	0.9035	0.7196	0.9008	4	0.2309	0.9191	0.7499	0.9611	4	0.1010	0.7058	0.5233	0.8873	2	0.7645	0.9037	0.4850	0.9613
C8orf58	3	0.5317	0.9035	0.5559	0.9008	7	0.8362	0.9191	0.7863	0.9611	8	0.6799	0.9054	0.6298	0.9044	5	0.4524	0.9037	0.2713	0.9257
CAB39L	1	0.7400	0.9035	0.7400	0.9008	3	0.3756	0.9191	0.8113	0.9611	4	0.7032	0.9054	0.7030	0.9051	4	0.6417	0.9037	0.9217	0.9975
CAMK2D	2	0.2462	0.9035	0.4553	0.9008	3	0.4125	0.9191	0.5114	0.9393	4	0.9447	0.9800	0.7543	0.9051	3	0.3309	0.8903	0.5047	0.9613
CASR	1	0.6461	0.9035	0.6461	0.9008	4	0.0276	0.9191	0.0012	0.2089	4	0.0457	0.7058	0.0029	0.4900	6	0.5906	0.9037	0.7926	0.9880
CATSPER3	3	0.2116	0.9035	0.1581	0.9008	6	0.7440	0.9191	0.4376	0.9393	8	0.5471	0.9054	0.4613	0.8873	7	0.3148	0.8903	0.4006	0.9494
CCAR2	6	0.3972	0.9035	0.5011	0.9008	9	0.5444	0.9191	0.2796	0.9393	12	0.9148	0.9800	0.2043	0.8873	9	0.2050	0.7538	0.2704	0.9257
CCDC36	4	0.1797	0.9035	0.1914	0.9008	5	0.4746	0.9191	0.2783	0.9393	9	0.1368	0.7058	0.2035	0.8873	4	0.1271	0.6353	0.2966	0.9306
CCDC62	2	0.3007	0.9035	0.4942	0.9008	5	0.8101	0.9191	0.6137	0.9393	6	0.2780	0.7624	0.3603	0.8873	7	0.0644	0.5420	0.0800	0.7225
CCDC71	1	0.6500	0.9035	0.6500	0.9008	4	0.2026	0.9191	0.8638	0.9611	5	0.1599	0.7260	0.8535	0.9251	6	0.5365	0.9037	0.6166	0.9880
CD19	2	0.7358	0.9035	0.8074	0.9055	5	0.9889	0.9898	0.1861	0.9393	7	0.9496	0.9800	0.2205	0.8873	NA	NA	NA	NA	NA
CHD9	6	0.8652	0.9087	0.6108	0.9008	16	0.5983	0.9191	0.5157	0.9393	20	0.5903	0.9054	0.9012	0.9363	5	0.1259	0.6353	0.2763	0.9257
CLCN3	1	0.7720	0.9035	0.7720	0.9008	5	0.5219	0.9191	0.5921	0.9393	5	0.5746	0.9054	0.5949	0.8873	2	0.9318	0.9630	0.6522	0.9880
COASY	3	0.1687	0.9035	0.4105	0.9008	5	0.2309	0.9191	0.9018	0.9712	8	0.9573	0.9800	0.7303	0.9051	4	0.6875	0.9037	0.8830	0.9880
CTSB	3	0.9743	0.9801	0.4239	0.9008	15	0.0279	0.9191	0.1788	0.9393	17	0.0688	0.7058	0.1153	0.8873	10	0.4903	0.9037	0.0664	0.7225
CUEDC2	1	0.8085	0.9035	0.8085	0.9055	1	0.1384	0.9191	0.1384	0.9393	2	0.3445	0.8269	0.4987	0.8873	2	0.6757	0.9037	0.9601	0.9975
CYLD	1	0.1195	0.9035	0.1195	0.9008	1	0.6384	0.9191	0.6384	0.9393	2	0.0925	0.7058	0.1786	0.8873	1	0.2440	0.8133	0.2440	0.9257
DALRD3	1	0.7229	0.9035	0.7229	0.9008	2	0.7994	0.9191	0.2768	0.9393	2	0.2633	0.7498	0.4053	0.8873	2	0.3829	0.9037	0.5877	0.9880
DDX46	1	0.7143	0.9035	0.7143	0.9008	2	0.3375	0.9191	0.7387	0.9611	3	0.2204	0.7484	0.6647	0.9051	NA	NA	NA	NA	NA
DEPDC1B	2	0.7495	0.9035	0.6505	0.9008	3	0.7822	0.9191	0.4048	0.9393	4	0.8330	0.9605	0.5747	0.8873	2	0.1620	0.7003	0.3204	0.9320
DLG2	6	0.7416	0.9035	0.2062	0.9008	4	0.9455	0.9647	0.2551	0.9393	8	0.6557	0.9054	0.8131	0.9060	6	0.5756	0.9037	0.4642	0.9522
DNAH17	32	0.1226	0.9035	0.7730	0.9008	62	0.3156	0.9191	0.4248	0.9393	85	0.0878	0.7058	0.5480	0.8873	95	0.6925	0.9037	0.7443	0.9880
DTX3L	1	0.6561	0.9035	0.6561	0.9008	5	0.2740	0.9191	0.5437	0.9393	6	0.2726	0.7624	0.5104	0.8873	11	0.0312	0.3836	0.0623	0.7225
DYRK1A	3	0.4255	0.9035	0.8983	0.9432	3	0.1039	0.9191	0.1039	0.9393	6	0.1428	0.7058	0.2814	0.8873	5	0.5944	0.9037	0.8688	0.9880
ELOVL3	1	0.4106	0.9035	0.4106	0.9008	2	0.9475	0.9647	0.2395	0.9393	2	0.6192	0.9054	0.1862	0.8873	6	0.2589	0.8180	0.2562	0.9257
ELOVL7	2	0.7509	0.9035	0.5681	0.9008	3	0.3589	0.9191	0.0963	0.9393	4	0.4656	0.9054	0.3589	0.8873	3	0.0351	0.3880	0.0287	0.5528
ERCC8	4	0.4423	0.9035	0.0878	0.9008	6	0.3146	0.9191	0.8180	0.9611	8	0.1475	0.7058	0.3481	0.8873	8	0.1759	0.7132	0.5947	0.9880
FAM162A	1	0.6855	0.9035	0.6855	0.9008	2	0.7994	0.9191	0.2768	0.9393	3	0.9248	0.9800	0.3201	0.8873	4	0.4329	0.9037	0.1604	0.9108
FAM184B	6	0.3790	0.9035	0.6500	0.9008	11	0.7454	0.9191	0.6520	0.9393	15	0.4394	0.9054	0.4792	0.8873	10	0.3513	0.9037	0.5939	0.9880
FBRSL1	6	0.9433	0.9663	0.5148	0.9008	6	0.6764	0.9191	0.3686	0.9393	9	0.8416	0.9605	0.8125	0.9060	4	0.7177	0.9037	0.9327	0.9975
FDFT1	3	0.7796	0.9035	0.2592	0.9008	5	0.4746	0.9191	0.2783	0.9393	8	0.7276	0.9054	0.2659	0.8873	2	0.4980	0.9037	0.7781	0.9880
GAK	6	0.2673	0.9035	0.3250	0.9008	22	0.6662	0.9191	0.2925	0.9393	26	0.9093	0.9800	0.2439	0.8873	24	1.0000	1.0000	0.7008	0.9880
GALC	4	0.8111	0.9035	0.0460	0.9008	5	0.0032	0.5335	0.0366	0.9393	8	0.0210	0.7058	0.0311	0.8873	7	0.2607	0.8180	0.8539	0.9880

GBF1	6	0.5526	0.9035	0.5486	0.9008	12	0.7912	0.9191	0.2612	0.9393	15	0.6914	0.9054	0.4504	0.8873	11	0.3763	0.9037	0.8750	0.9880
GIN1	2	0.0979	0.9035	0.0979	0.9008	3	0.4592	0.9191	0.4372	0.9393	5	0.8408	0.9605	0.3471	0.8873	4	0.5775	0.9037	0.6325	0.9880
GPATCH8	7	0.4630	0.9035	0.0859	0.9008	13	0.4158	0.9191	0.9470	0.9881	17	0.2503	0.7498	0.2075	0.8873	15	0.6928	0.9037	0.4202	0.9494
GPNMB	5	0.1146	0.9035	0.1666	0.9008	9	0.4736	0.9191	0.4876	0.9393	10	0.1090	0.7058	0.1505	0.8873	19	0.6353	0.9037	0.6011	0.9880
GPR65	1	0.2106	0.9035	0.2106	0.9008	1	0.6384	0.9191	0.6384	0.9393	2	0.1980	0.7484	0.3930	0.8873	1	0.8401	0.9037	0.8401	0.9880
GRN	4	0.8086	0.9035	0.7977	0.9055	5	0.2677	0.9191	0.1369	0.9393	8	0.2562	0.7498	0.3408	0.8873	14	0.8368	0.9037	0.8802	0.9880
GUSB	1	0.2409	0.9035	0.2409	0.9008	5	0.8101	0.9191	0.6137	0.9393	5	0.6942	0.9054	0.1613	0.8873	2	0.9750	0.9936	0.4085	0.9494
HIP1R	3	0.8451	0.9046	0.9205	0.9487	12	0.6077	0.9191	0.4149	0.9393	14	0.7842	0.9419	0.4913	0.8873	18	0.3316	0.8903	0.7686	0.9880
HIST1H1B	2	0.7274	0.9035	0.7769	0.9008	7	0.6789	0.9191	0.4953	0.9393	8	0.6964	0.9054	0.4938	0.8873	5	0.2120	0.7538	0.3566	0.9494
HSD17B1	1	0.6689	0.9035	0.6689	0.9008	3	0.1506	0.9191	0.1660	0.9393	3	0.2230	0.7484	0.1730	0.8873	4	0.7090	0.9037	0.9326	0.9975
HSD3B7	4	0.2818	0.9035	0.1441	0.9008	6	0.4715	0.9191	0.3971	0.9393	9	0.2063	0.7484	0.1742	0.8873	8	0.4711	0.9037	1.0000	1.0000
IGF2BP3	1	0.7171	0.9035	0.7171	0.9008	3	0.8949	0.9503	0.2518	0.9393	4	0.5992	0.9054	0.3718	0.8873	2	0.3860	0.9037	0.2285	0.9257
INPP5F	6	0.3840	0.9035	0.1312	0.9008	7	0.9898	0.9898	0.4517	0.9393	12	0.6956	0.9054	0.3527	0.8873	10	0.2091	0.7538	0.8206	0.9880
INTS2	3	0.8479	0.9046	0.9327	0.9488	9	0.6533	0.9191	0.5168	0.9393	11	0.5708	0.9054	0.8194	0.9060	8	0.2329	0.7981	0.3851	0.9494
IP6K2	1	0.3997	0.9035	0.3997	0.9008	4	0.9072	0.9526	0.6541	0.9393	4	0.8666	0.9643	0.6972	0.9051	2	0.4053	0.9037	0.1708	0.9108
ITGA2B	1	0.4883	0.9035	0.4883	0.9008	11	0.0302	0.9191	0.5151	0.9393	11	0.0996	0.7058	0.9571	0.9731	8	0.4488	0.9037	0.9803	1.0000
ITGA8	2	0.1473	0.9035	0.1106	0.9008	10	0.1905	0.9191	0.7999	0.9611	12	0.0533	0.7058	0.4553	0.8873	18	0.6969	0.9037	0.8701	0.9880
ITPKB	3	0.8370	0.9046	0.9181	0.9487	10	0.1291	0.9191	0.9691	0.9944	12	0.1774	0.7355	0.9632	0.9731	11	0.0242	0.3836	0.3976	0.9494
JADE2	3	0.8859	0.9244	0.9526	0.9582	1	0.5191	0.9191	0.5191	0.9393	4	0.6243	0.9054	0.8822	0.9309	6	0.7882	0.9037	0.9406	0.9975
KCNIP3	2	0.4888	0.9035	0.7290	0.9008	5	0.4282	0.9191	0.2800	0.9393	6	0.3086	0.7624	0.2935	0.8873	2	0.3692	0.9037	0.8689	0.9880
KCNN3	2	0.4135	0.9035	0.8875	0.9377	1	0.3375	0.9191	0.3375	0.9393	3	0.2094	0.7484	0.6407	0.9051	1	0.5280	0.9037	0.5280	0.9822
KCNS3	1	0.5806	0.9035	0.5806	0.9008	5	0.5739	0.9191	0.4703	0.9393	6	0.4764	0.9054	0.5432	0.8873	10	0.6460	0.9037	0.9775	1.0000
KLHL7	1	0.3222	0.9035	0.3222	0.9008	1	0.6384	0.9191	0.6384	0.9393	2	0.7213	0.9054	0.4929	0.8873	NA	NA	NA	NA	NA
KRTCAP2	3	0.7635	0.9035	0.9306	0.9488	2	0.7994	0.9191	0.2768	0.9393	5	0.4789	0.9054	0.5837	0.8873	1	0.8440	0.9037	0.8440	0.9880
LAMB2	9	0.1353	0.9035	0.0445	0.9008	13	0.2188	0.9191	0.6719	0.9567	19	0.6635	0.9054	0.1355	0.8873	30	0.7479	0.9037	0.6907	0.9880
LAT	2	0.7478	0.9035	0.8498	0.9152	4	0.2026	0.9191	0.8638	0.9611	5	0.3500	0.8281	0.8719	0.9271	3	0.8432	0.9037	0.4123	0.9494
LCORL	2	0.7586	0.9035	0.2459	0.9008	4	0.7898	0.9191	0.5175	0.9393	6	0.7736	0.9418	0.3650	0.8873	2	0.4616	0.9037	0.9017	0.9975
LRRK2	7	0.9041	0.9376	0.3714	0.9008	20	0.9461	0.9647	0.4681	0.9393	23	0.9625	0.9800	0.3020	0.8873	43	0.0002	0.0328	0.0017	0.2205
LRRN4	1	0.8228	0.9046	0.8228	0.9056	8	0.0552	0.9191	0.7655	0.9611	8	0.1288	0.7058	0.8866	0.9309	9	0.6428	0.9037	0.0195	0.5193
MAL	2	0.7023	0.9035	0.6243	0.9008	2	0.2158	0.9191	0.3889	0.9393	3	0.3978	0.8911	0.2859	0.8873	3	0.0114	0.3045	0.0161	0.5158
MALSU1	2	0.3381	0.9035	0.8302	0.9056	1	0.6384	0.9191	0.6384	0.9393	3	0.1795	0.7355	0.5981	0.8873	3	0.3309	0.8903	0.5896	0.9880
MCCC1	3	0.7986	0.9035	0.0945	0.9008	6	0.1232	0.9191	0.9293	0.9876	8	0.1268	0.7058	0.4922	0.8873	16	0.6551	0.9037	0.4513	0.9522
MED12L	2	0.3580	0.9035	0.5381	0.9008	10	0.2561	0.9191	0.9390	0.9876	12	0.1361	0.7058	0.8657	0.9271	7	0.6451	0.9037	0.9183	0.9975
MED13	11	0.4649	0.9035	0.4516	0.9008	17	0.1719	0.9191	0.1428	0.9393	24	0.1267	0.7058	0.3488	0.8873	20	1.0000	1.0000	0.0808	0.7225
MLX	1	0.7229	0.9035	0.7229	0.9008	1	0.6384	0.9191	0.6384	0.9393	2	0.8001	0.9533	0.5359	0.8873	1	0.6286	0.9037	0.6286	0.9880
MMRN1	8	0.1398	0.9035	0.3741	0.9008	22	0.7883	0.9191	0.1454	0.9393	22	0.2319	0.7492	0.4890	0.8873	23	0.2938	0.8705	0.4281	0.9494
MRPS5	4	0.2224	0.9035	0.2238	0.9008	4	0.5231	0.9191	0.7161	0.9611	6	0.5053	0.9054	0.6671	0.9051	6	0.7877	0.9037	1.0000	1.0000
NAGLU	1	0.6735	0.9035	0.6735	0.9008	3	0.3756	0.9191	0.8113	0.9611	4	0.5930	0.9054	0.7897	0.9060	3	0.0056	0.2263	0.0290	0.5528
NCAPG	1	0.1429	0.9035	0.1429	0.9008	10	0.1494	0.9191	0.8286	0.9611	10	0.6500	0.9054	0.7402	0.9051	14	0.4414	0.9037	0.4144	0.9494
NCKIPSD	4	0.4901	0.9035	0.5815	0.9008	10	0.1678	0.9191	0.9857	0.9975	11	0.6280	0.9054	0.9156	0.9437	8	0.7699	0.9037	0.9043	0.9975
NDUFAF2	1	0.4148	0.9035	0.4148	0.9008	2	0.3375	0.9191	0.7387	0.9611	2	0.9586	0.9800	0.8294	0.9060	NA	NA	NA	NA	NA
NEK1	4	0.5022	0.9035	0.6610	0.9008	11	0.3849	0.9191	0.7447	0.9611	13	0.2862	0.7624	0.5303	0.8873	12	0.7178	0.9037	0.8595	0.9880
NFATC2IP	3	0.4214	0.9035	0.7313	0.9008	1	0.7822	0.9191	0.7822	0.9611	3	0.5211	0.9054	0.5588	0.8873	3	0.4512	0.9037	0.3567	0.9494
NFKB2	1	0.0180	0.9035	0.0180	0.9008	7	0.2543	0.9191	0.9714	0.9944	7	0.0333	0.7058	0.0381	0.8873	6	0.7477	0.9037	0.2684	0.9257
NMD3	1	0.7111	0.9035	0.7111	0.9008	4	0.7898	0.9191	0.5175	0.9393	5	0.7570	0.9351	0.4835	0.8873	5	0.5480	0.9037	0.7658	0.9880
NOD2	8	0.5319	0.9035	0.4441	0.9008	19	0.8268	0.9191	0.1938	0.9393	23	0.6156	0.9054	0.1243	0.8873	27	0.0995	0.6353	0.2063	0.9257
NOL4	3	0.3726	0.9035	0.1681	0.9008	2	0.6369	0.9191	0.1488	0.9393	4	0.3045	0.7624	0.3906	0.8873	NA	NA	NA	NA	NA
NOLC1	3	0.4175	0.9035	0.7828	0.9008	2	0.3375	0.9191	0.7387	0.9611	5	0.7251	0.9054	0.7730	0.9060	6	0.8421	0.9037	0.4792	0.9613
NUCKS1	2	0.8655	0.9087	0.8655	0.9261	1	0.1384	0.9191	0.1384	0.9393	2	0.4248	0.9054	0.1326	0.8873	NA	NA	NA	NA	NA
NUPL2	3	0.8508	0.9046	0.9582	0.9582	3	0.6929	0.9191	0.8897	0.9643	4	0.8462	0.9605	0.9731	0.9731	11	0.0633	0.5420	0.0813	0.7225
OR2B6	2	0.4246	0.9035	0.6812	0.9008	2	0.5210	0.9191	0.6522	0.9393	3	0.2615	0.7498	0.5370	0.8873	8	0.5192	0.9037	0.8471	0.9880
P4HTM	4	0.0284	0.9035	0.3338	0.9008	6	0.7669	0.9191	0.6401	0.9393	8	0.3925	0.8911	0.4503	0.8873	4	0.6599	0.9037	0.7552	0.9880
PAM	5	0.4430	0.9035	0.1454	0.9008	13	0.2178	0.9191	0.2638	0.9393	17	0.1331	0.7058	0.1188	0.8873	11	0.5297	0.9037	0.8316	0.9880

PARP9	3	0.0819	0.9035	0.1087	0.9008	9	0.0730	0.9191	0.1820	0.9393	11	0.0110	0.7058	0.0215	0.8873	7	0.0908	0.6353	0.0311	0.5528
PBXIP1	1	0.3142	0.9035	0.3142	0.9008	8	0.0656	0.9191	0.1180	0.9393	9	0.0457	0.7058	0.1603	0.8873	10	0.6148	0.9037	0.2516	0.9257
PDLIM2	2	0.3483	0.9035	0.8414	0.9120	5	0.4616	0.9191	0.9406	0.9876	6	0.9496	0.9800	0.7934	0.9060	5	0.1135	0.6353	0.3103	0.9320
PGF	1	0.6279	0.9035	0.6279	0.9008	1	0.6384	0.9191	0.6384	0.9393	2	0.9789	0.9847	0.6894	0.9051	1	0.7501	0.9037	0.7501	0.9880
PGS1	1	0.2494	0.9035	0.2494	0.9008	6	0.5953	0.9191	0.6489	0.9393	7	0.4686	0.9054	0.5630	0.8873	3	0.2932	0.8705	0.1627	0.9108
PLEKHH3	2	0.3327	0.9035	0.7597	0.9008	5	0.8603	0.9265	0.5964	0.9393	6	0.4497	0.9054	0.7379	0.9051	3	0.6548	0.9037	0.6322	0.9880
PMVK	1	0.2167	0.9035	0.2167	0.9008	2	0.5996	0.9191	0.4135	0.9393	2	0.2109	0.7484	0.4153	0.8873	6	0.3659	0.9037	0.3828	0.9494
POLR2A	1	0.7451	0.9035	0.7451	0.9008	4	0.4592	0.9191	0.1869	0.9393	5	0.1749	0.7355	0.2302	0.8873	1	0.1190	0.6353	0.1190	0.8658
PPIP5K2	1	0.7418	0.9035	0.7418	0.9008	7	0.9172	0.9571	0.3561	0.9393	7	0.8111	0.9596	0.5055	0.8873	4	0.3778	0.9037	0.6076	0.9880
PPM1L	2	0.2739	0.9035	0.5785	0.9008	2	0.8548	0.9265	0.8548	0.9611	2	0.5182	0.9054	0.7981	0.9060	1	0.1031	0.6353	0.1031	0.8463
PROM2	3	0.1249	0.9035	0.2403	0.9008	6	0.3360	0.9191	0.5467	0.9393	8	0.1312	0.7058	0.1945	0.8873	15	0.0186	0.3836	0.1504	0.9108
PROX2	2	0.7240	0.9035	0.7255	0.9008	9	0.7023	0.9191	0.4736	0.9393	9	0.7206	0.9054	0.2837	0.8873	6	0.9004	0.9416	0.9941	1.0000
PRSS3	2	0.7655	0.9035	0.2086	0.9008	12	0.6754	0.9191	1.0000	1.0000	12	0.7029	0.9054	0.8698	0.9271	NA	NA	NA	NA	NA
PRSS53	1	0.1227	0.9035	0.1227	0.9008	5	0.6002	0.9191	0.4121	0.9393	6	0.1784	0.7355	0.2587	0.8873	8	0.7765	0.9037	0.9536	0.9975
PSD	1	0.6511	0.9035	0.6511	0.9008	7	0.5755	0.9191	0.8112	0.9611	8	0.4628	0.9054	0.7714	0.9060	9	0.1455	0.6920	0.4451	0.9522
QARS	6	0.8305	0.9046	0.7614	0.9008	12	0.2576	0.9191	0.9766	0.9944	17	0.4203	0.9054	0.9573	0.9731	10	0.1514	0.6920	0.6430	0.9880
RAB29	1	0.6480	0.9035	0.6480	0.9008	1	0.6384	0.9191	0.6384	0.9393	2	0.9754	0.9847	0.6675	0.9051	2	0.0193	0.3836	0.0469	0.6816
RABEP2	2	0.6963	0.9035	0.5990	0.9008	3	0.7822	0.9191	0.4048	0.9393	5	0.6014	0.9054	0.5147	0.8873	2	0.8496	0.9037	0.5452	0.9880
RABGEF1	4	0.8040	0.9035	0.8057	0.9055	4	0.7898	0.9191	0.5175	0.9393	8	0.8668	0.9643	0.5972	0.8873	2	0.1190	0.6353	0.1190	0.8658
RIMS1	2	0.7112	0.9035	0.7487	0.9008	8	0.1424	0.9191	0.3517	0.9393	9	0.1869	0.7475	0.3288	0.8873	9	0.0900	0.6353	0.2154	0.9257
RIT2	2	0.7513	0.9035	0.5623	0.9008	1	0.6384	0.9191	0.6384	0.9393	3	0.8211	0.9605	0.5254	0.8873	2	0.1833	0.7132	0.4331	0.9494
RPS6KL1	2	0.7013	0.9035	0.7402	0.9008	3	0.0209	0.9191	0.1316	0.9393	5	0.0901	0.7058	0.3114	0.8873	2	0.3915	0.9037	0.6685	0.9880
SCAF11	6	0.5627	0.9035	0.6599	0.9008	15	0.6230	0.9191	0.3785	0.9393	19	0.8523	0.9610	0.5607	0.8873	19	0.5245	0.9037	0.9572	0.9975
SCARB2	2	0.0833	0.9035	0.1983	0.9008	3	0.7822	0.9191	0.4048	0.9393	4	0.1375	0.7058	0.0829	0.8873	11	1.0000	1.0000	0.5043	0.9613
SEC23IP	3	0.0276	0.9035	0.1313	0.9008	9	0.0332	0.9191	0.1113	0.9393	11	0.0040	0.6669	0.0469	0.8873	10	0.6819	0.9037	0.3037	0.9320
SEC24A	5	0.3807	0.9035	0.6596	0.9008	6	0.1474	0.9191	0.8698	0.9614	10	0.0939	0.7058	0.7062	0.9051	11	0.8046	0.9037	0.1658	0.9108
SETD1A	6	0.8372	0.9046	0.3355	0.9008	8	0.5726	0.9191	0.2707	0.9393	14	0.4997	0.9054	0.2982	0.8873	11	0.1872	0.7132	0.2870	0.9257
SH2B1	4	0.1085	0.9035	0.5687	0.9008	6	0.5974	0.9191	0.6517	0.9393	9	0.1351	0.7058	0.3903	0.8873	5	0.1210	0.6353	0.3183	0.9320
SH3GL2	2	0.4685	0.9035	0.7186	0.9008	4	0.4115	0.9191	0.2129	0.9393	5	0.2248	0.7484	0.2218	0.8873	3	0.3707	0.9037	0.3626	0.9494
SH3RF1	3	0.5206	0.9035	0.8288	0.9056	8	0.3401	0.9191	0.8124	0.9611	10	0.2467	0.7498	0.7886	0.9060	4	0.8078	0.9037	0.8332	0.9880
SIPA1L2	9	0.7385	0.9035	0.7701	0.9008	19	0.2038	0.9191	1.0000	1.0000	23	0.4074	0.9005	0.9685	0.9731	20	0.0557	0.5420	0.2218	0.9257
SLC18B1	5	0.7878	0.9035	0.5817	0.9008	4	0.5912	0.9191	0.9354	0.9876	7	0.5473	0.9054	0.7932	0.9060	11	0.5968	0.9037	1.0000	1.0000
SLC25A20	1	0.6936	0.9035	0.6936	0.9008	2	0.7994	0.9191	0.2768	0.9393	3	0.2954	0.7624	0.3154	0.8873	2	0.3339	0.8903	0.4909	0.9613
SLC45A3	4	0.9876	0.9876	0.3251	0.9008	4	0.6615	0.9191	0.9564	0.9919	6	0.7052	0.9054	0.7396	0.9051	11	0.0289	0.3836	0.4573	0.9522
SLC4A1	4	0.5729	0.9035	0.4211	0.9008	5	0.4590	0.9191	0.3622	0.9393	8	0.2496	0.7498	0.6941	0.9051	12	0.1143	0.6353	0.6278	0.9880
SLC50A1	2	0.7567	0.9035	0.5072	0.9008	1	0.6384	0.9191	0.6384	0.9393	3	0.9143	0.9800	0.5121	0.8873	2	0.2344	0.7981	0.2555	0.9257
SNX20	2	0.9611	0.9785	0.0389	0.9008	1	0.2026	0.9191	0.2026	0.9393	2	0.5690	0.9054	0.6715	0.9051	2	0.3986	0.9037	0.4583	0.9522
SPNS1	3	0.8121	0.9035	0.7559	0.9008	6	0.8419	0.9191	0.3711	0.9393	8	0.6362	0.9054	0.3524	0.8873	3	0.2911	0.8705	0.2369	0.9257
STK39	2	0.6269	0.9035	0.1610	0.9008	1	0.6384	0.9191	0.6384	0.9393	3	0.6681	0.9054	0.3160	0.8873	1	0.8081	0.9037	0.8081	0.9880
SYT17	3	0.4194	0.9035	0.2076	0.9008	9	0.3629	0.9191	0.8546	0.9611	11	0.6318	0.9054	0.5587	0.8873	6	0.0300	0.3836	0.1465	0.9108
SYT4	1	0.6320	0.9035	0.6320	0.9008	3	0.1039	0.9191	0.1039	0.9393	4	0.3084	0.7624	0.1972	0.8873	5	0.9329	0.9630	0.8581	0.9880
TMEM175	2	0.4424	0.9035	0.4785	0.9008	11	0.5727	0.9191	0.5181	0.9393	11	0.7221	0.9054	0.5466	0.8873	15	0.9448	0.9690	0.1479	0.9108
TOX3	4	0.2977	0.9035	0.3371	0.9008	7	0.2156	0.9191	0.3371	0.9393	8	0.5008	0.9054	0.3575	0.8873	6	0.0293	0.3836	0.0433	0.6816
TRIM10	2	0.7265	0.9035	0.7811	0.9008	5	0.8424	0.9191	0.5701	0.9393	7	0.7057	0.9054	0.6049	0.8873	6	0.5480	0.9037	0.7599	0.9880
TRIM31	1	0.7195	0.9035	0.7195	0.9008	6	0.9327	0.9647	0.4383	0.9393	7	0.8830	0.9696	0.4592	0.8873	14	0.0105	0.3045	0.1058	0.8463
TUFM	1	0.3609	0.9035	0.3609	0.9008	2	0.3756	0.9191	0.5208	0.9393	2	0.0840	0.7058	0.1205	0.8873	1	0.1826	0.7132	0.1826	0.9257
TXNDC15	1	0.1447	0.9035	0.1447	0.9008	1	0.6384	0.9191	0.6384	0.9393	2	0.1206	0.7058	0.2376	0.8873	3	0.4744	0.9037	0.8499	0.9880
UBAP2	5	0.2782	0.9035	0.3420	0.9008	13	0.3453	0.9191	0.3691	0.9393	18	0.1152	0.7058	0.3504	0.8873	23	0.0024	0.1939	0.0602	0.7225
UBTF	1	0.2096	0.9035	0.2096	0.9008	3	0.3756	0.9191	0.8113	0.9611	4	0.7850	0.9419	0.6092	0.8873	3	0.6214	0.9037	0.8585	0.9880
USP19	4	0.2391	0.9035	0.6810	0.9008	10	0.7478	0.9191	0.7022	0.9611	14	0.4296	0.9054	0.6843	0.9051	14	0.1609	0.7003	0.7528	0.9880
USP4	3	0.6123	0.9035	0.6026	0.9008	10	0.9654	0.9770	0.6022	0.9393	11	0.7223	0.9054	0.3998	0.8873	11	0.0969	0.6353	0.0045	0.2418
VKORC1	2	0.6118	0.9035	0.1532	0.9008	1	0.7822	0.9191	0.7822	0.9611	2	0.4903	0.9054	0.3075	0.8873	1	0.3309	0.8903	0.3309	0.9455
VPS13C	26	0.2533	0.9035	0.4155	0.9008	24	0.1276	0.9191	0.4043	0.9393	42	0.0557	0.7058	0.4313	0.8873	49	0.0636	0.5420	0.1573	0.9108

WDHD1	12	0.5873	0.9035	0.2211	0.9008	11	0.4654	0.9191	0.4806	0.9393	19	0.3677	0.8580	0.2210	0.8873	17	0.1859	0.7132	0.7812	0.9880
WDR5B	1	0.7811	0.9035	0.7811	0.9008	4	0.3299	0.9191	0.8181	0.9611	4	0.3043	0.7624	0.7500	0.9051	5	0.4553	0.9037	0.8421	0.9880
WDR6	4	0.9196	0.9478	0.2073	0.9008	5	0.4746	0.9191	0.2783	0.9393	9	0.6897	0.9054	0.3304	0.8873	14	0.0364	0.3880	0.3782	0.9494
YLPM1	6	0.1770	0.9035	0.3788	0.9008	16	0.7444	0.9191	0.8602	0.9611	20	0.2272	0.7484	0.5507	0.8873	4	0.0835	0.6353	0.0703	0.7225
ZBTB7B	1	0.4156	0.9035	0.4156	0.9008	4	0.2309	0.9191	0.7499	0.9611	5	0.6445	0.9054	0.5919	0.8873	3	0.5584	0.9037	0.4289	0.9494
ZKSCAN8	1	0.7111	0.9035	0.7111	0.9008	4	0.4592	0.9191	0.1869	0.9393	5	0.1967	0.7484	0.2512	0.8873	7	0.6537	0.9037	0.7423	0.9880
ZNF165	1	0.7483	0.9035	0.7483	0.9008	2	0.3756	0.9191	0.5208	0.9393	2	0.4532	0.9054	0.6815	0.9051	4	0.6998	0.9037	0.5590	0.9880
ZNF514	4	0.5021	0.9035	0.6431	0.9008	3	0.7341	0.9191	0.8860	0.9643	7	0.8816	0.9696	0.8101	0.9060	7	0.8110	0.9037	0.8439	0.9880
ZNF629	1	0.2161	0.9035	0.2161	0.9008	4	0.4592	0.9191	0.1869	0.9393	5	0.1512	0.7058	0.1987	0.8873	6	0.5380	0.9037	0.7009	0.9880
ZNF646	4	0.6189	0.9035	0.1858	0.9008	21	0.2231	0.9191	0.5886	0.9393	25	0.1485	0.7058	0.4446	0.8873	18	0.8529	0.9037	0.6163	0.9880
ZNF668	1	0.2441	0.9035	0.2441	0.9008	3	0.7822	0.9191	0.4048	0.9393	4	0.4570	0.9054	0.3489	0.8873	2	0.6070	0.9037	0.4985	0.9613
ZSCAN16	1	0.6212	0.9035	0.6212	0.9008	4	0.0499	0.9191	0.1794	0.9393	5	0.0882	0.7058	0.1388	0.8873	4	0.7037	0.9037	0.8740	0.9880

NA: not available, used where the gene in question had no variants that fit the inclusion criteria in the given dataset
