# Supplementary material for: Meta-analysis of whole-exome sequencing data from two independent cohorts finds no evidence for rare variant enrichment in Parkinson disease associated loci
Source: PLoS One. 2020 Oct 1;15(10):e0239824. doi: 10.1371/journal.pone.0239824 (PMC7529297; doi:10.1371/journal.pone.0239824)
Supplement: S3 Table — (PDF) [file pone.0239824.s004.pdf]

S3 Table - Gene set analyses

	ParkWest			PPMI			Meta			NeuroX		
**Variant subset**	Variants	Burden P	SKAT P	Variants	Burden P	SKAT P	Variants	Burden P	SKAT P	Variants	Burden P	SKAT P
NSSS	554	0.798	0.444	1341	0.2825	0.5254	1707	0.3983	0.3706	1543	0.827	0.0705
LoF	14	0.1199	0.1152	17	0.4218	0.7841	30	0.1144	0.421	40	0.141	0.5268

NSSS: nonsynonymous, stop-gain, stop-los and splicing variants; LoF: loss-of-function variants. All p-values are nominal, and not corrected for multiple testing in this table
